# Spontaneous Activity Associated with Delusions of Schizophrenia in the Left Medial Superior Frontal Gyrus: A Resting-State fMRI Study

**DOI:** 10.1371/journal.pone.0133766

**Published:** 2015-07-23

**Authors:** Bin Gao, Yiquan Wang, Weibo Liu, Zhiyu Chen, Heshan Zhou, Jinyu Yang, Zachary Cohen, Yihong Zhu, Yufeng Zang

**Affiliations:** 1 Department of Psychiatry, the Second Affiliated Hospital, School of Medicine, Zhejiang University, Hangzhou, Zhejiang, PR China; 2 Hangzhou Seventh People's Hospital, Hangzhou, Zhejiang, PR China; 3 Hangzhou First People's Hospital, Hangzhou, Zhejiang, PR China; 4 Department of Public Health, School of Medicine, Zhejiang University, Hangzhou, Zhejiang, PR China; 5 Alpert Medical School of Brown University, Richmond St., Providence, RI, United States of America; 6 Mental Health Education and Counseling Center, Zhejiang University, Hangzhou, Zhejiang, PR China; 7 Center for Cognition and Brain Disorders, Hangzhou Normal University, Hangzhou, Zhejiang, PR China; 8 Zhejiang Key Laboratory for Research in Assessment of Cognitive Impairments, Hangzhou, Zhejiang, PR China; 9 Mental Health Center, School of Medicine, Zhe Jiang University, Hangzhou, Zhejiang, PR China; Yale University School of Medicine, UNITED STATES

## Abstract

Delusions of schizophrenia have been found to be associated with alterations of some brain regions in structure and task-induced activation. However, the relationship between spontaneously occurring symptoms and spontaneous brain activity remains unclear. In the current study, 14 schizophrenic patients with delusions and 14 healthy controls underwent a resting-state functional magnetic resonance imaging (RS-fMRI) scan. Patients with delusions of schizophrenia patients were rated with Positive and Negative Syndrome Scale (PANSS) and Characteristics of Delusional Rating Scale (CDRS). Regional homogeneity (ReHo) was calculated to measure the local synchronization of the spontaneous activity in a voxel-wise way. A two-sample t-test showed that ReHo of the right anterior cingulate gyrus and left medial superior frontal gyrus were higher in patients, and ReHo of the left superior occipital gyrus was lower, compared to healthy controls. Further, among patients, correlation analysis showed a significant difference between delusion scores of CRDS and ReHo of brain regions. ReHo of the left medial superior frontal gyrus was negatively correlated with patients’ CDRS scores but not with delusional PANSS scores. These results suggested that altered local synchronization of spontaneous brain activity may be related to the pathophysiology of delusion in schizophrenia.

## Introduction

“Delusion is a false belief based on incorrect inference about external reality that is firmly sustained despite what almost everyone else believes and despite what constitutes incontrovertible and obvious proof of evidence to the contrary” [American Psychiatric Association (APA), 1994]. Delusion is a core symptom for the diagnosis of schizophrenia [1], occurring in more than 70% of schizophrenia patients [2]. Identifying the neuroanatomical and functional underpinnings of specific symptoms offers significant insight into the etiology of schizophrenia [3].

Some evidence has been accumulated from structural magnetic resonance imaging (MRI) and task-related functional MRI (fMRI) studies. Structure alterations related to delusions in schizophrenia have been explored [4–12]. In previous studies, Whitford and his colleagues found that schizophrenic patients' delusion severity was positively correlated with the volume of the dorso-medial prefrontal cortex (DMPFC), centered on the medial frontal gyrus [13]. Although many structurally-altered brain areas were related to delusions of schizophrenia, structural brain changes usually occurred in relatively later stages of schizophrenia patients. Brain function research has been applied in the past to explore earlier brain changes, Many fMRI studies investigated brain activation in schizophrenic patients with delusions using cognitive tasks, such as a feedback task which was used to investigate the neural responses to feedback of (successful vs. unsuccessful) monetary gain or avoidance of loss [14]. A reference evoking task was also used in these studies, which was carried out by viewing video vignettes of referential conversations, non-referential conversations or no conversations between two people, filmed at varying distances of 1 m, 5 m or 10 m, while undergoing an fMRI scan [15]. A working memory task including auditory and visual memory was employed as well [16]. The severity of delusions in schizophrenic patients was negatively correlated with activation in the superior temporal sulcus in a reference-evoking task, which might be related to the formation of referential or persecutory delusions, and activation of the superior temporal sulcus might affect the severity of delusion [15]. However, the delusions in schizophrenic patients are spontaneous and it is difficult to design a specific task that can evoke delusions. Therefore, to explore the association of spontaneous brain activity and delusions may be helpful to reveal the underlying brain activity of delusions.

Positron emission tomography (PET) and resting-state fMRI (RS-fMRI) are important instruments to explore spontaneous brain activity. PET studies have shown the association between delusions and resting-state brain activity. For example, it was found that delusions showed a strong negative correlation with regional cerebral blood flow (rCBF) in the left frontal cortex [17] and left parahippocampal gyrus [18]. However, compared to PET, RS-fMRI does not require intravenous injection and has better temporal and spatial resolution. An increasing number of RS-fMRI studies have been carried out to investigate spontaneous activity since the first RS-fMRI report by Biswal and colleagues [19]. Most RS-fMRI studies have investigated the synchronization or functional connectivity of the time courses among “distinct” brain regions [20–26]; however regional homogeneity (ReHo) [27] has been used to analyze “local” synchronization. An abnormal functional connectivity between two distinct regions indicates an abnormal relationship between these two regions; however, the method does not confirm which specific region is abnormal. In contrast, an abnormal ReHo indicates abnormal brain activity in a specific region. For example, abnormally increased ReHo in the medial temporal lobe has been reported in medial temporal lobe epilepsy (mTLE) patients [28–30], suggesting increased neuronal local synchronization in the epileptic focus. ReHo assumes that, within a functional cluster, the hemodynamic feature of each voxel would be similar or synchronous with that of others, and that this similarity could be altered or modulated in different situations. Higher ReHo indicates more synchronization in the functional cluster, which may illustrate functional synchronization in certain brain regions [27]. This method has already been used for investigation of functional modulations in the resting state for patients with schizophrenia [31, 32], Alzheimer’s disease (AD) [33], Parkinson’s disease (PD) [34], and other pathological states (see review by [35, 36]).

In this study, we were interested in whether schizophrenic patients with delusions would show abnormal ReHo and, if so, whether the brain areas with abnormal ReHo were correlated with delusions.

## Methods

### 2.1 Participants

Fourteen right-handed schizophrenia inpatients and outpatients of the Seventh People’s Hospital of Hangzhou were included in the study. All patients were diagnosed as paranoid schizophrenics according to DSM-IV (American Psychiatric Association, 1994) and the delusions were the predominant symptom of each patient; i.e., all patients were experiencing stable delusions during the experiment. Each patient had a single delusion. The most prevalent delusion was persecution delusion, which also included reference and guilt delusion. All the patients were assessed and diagnosed by experienced psychiatrists of the Seventh People’s Hospital of Hangzhou, using structured clinical interview for DSM-IV (SCID). The Positive and Negative Syndrome Scale (PANSS) [37] and Characteristics of Delusional Rating Scale (CDRS) [38] were used as instruments of clinical assessment. CDRS is a widely used multidimensional instrument for evaluating delusions [1] and is regarded as an expert rating scale of delusion [1]. It contains 11 dimensions: conviction, preoccupation, interference, resistance, dismissibility, absurdity, self-evidentness, reassurance, worry, unhappiness and pervasiveness [38]. All patients’ scales were rated by a trained and experienced psychiatrist. One patient missed one item (pervasiveness) in the CDRS. We used the mean CDRS score (total score divided by the number of items) to avoid the influence of the missing item. Fourteen right-handed healthy controls were recruited from Hangzhou by advertisements posted in the Hangzhou community. All healthy controls were also assessed by the psychiatrists of the Seventh People’s Hospital of Hangzhou, using the structured clinical interview according to the DSM-IV (SCID). None had psychosis. The two groups were matched for age, gender, and education level (Table 1).

**Table 1:** Demographic and clinical parameters of healthy control and schizophrenic patient groups (Mean ± SD).

	Group	
	Schizophrenia patients	Healthy controls
Gender (M/F)	9/5	9/5
Age (years)	33.2±10.7	34.9±13.6
Education (years)	11.7±2.7	11.3±2.3
Illness duration (years)	9.2±8.5	N/A
Medication (average chlorpromazine equivalent, mg/day)	586.5±327	N/A
CDRS (average)	6.2±1.6	N/A
PANSS		N/A
Total	74.1±16.2	
Positive	16.4±5.3	
Delusions(P1)	3.9±1.2	
Negative	22.6±6.2	
General	30.8±8.7	

N/A: Not applicable. P1: The item of PANSS-Delusion subscale.

Exclusion criteria for participants were i) a history of head injury, neurological disease such as epilepsy, other serious illness, alcohol dependence, exposure to electroconvulsive therapy, and other psychiatric disorders (healthy controls with a history of schizophrenia and a family history of psychosis were also excluded), and ii) the intake of medication in 6 hours before the fMRI scan.

This study was approved by the ethics committee of the Seventh People’s Hospital of Hangzhou and Zhejiang University. We gave each participant or the legally authorized representative detailed information on the processes (including possible risks) of the study with commonly used words until that he/she understood completely. When he/she agreed, he/she would sign an informed consent. Written informed consent of the schizophrenic patient was obtained from his/her legally authorized representative and the control provided written informed consent himself/herself.

### 2.2 Image acquisition

Each participant was scanned with a 1.5 Tesla Siemens Sonata scanner. Foam pads were used to reduce head motion. The functional T2*-weighted resting-state images were acquired using an echo planar imaging (EPI) sequence (TR/TE 2000/40 ms, FA 90°, FOV 240 × 240 mm, matrix 64 × 64, slice thickness 5 mm with 1 a mm gap, 23 slices, scan time 8 min, 240 volumes). The participants were instructed to lie still with their eyes closed and not to think of anything in particular during RS-fMRI data acquisition [19, 27, 39].

### 2.3 Data analysis

#### 2.3.1 ReHo calculation

The fMRI data preprocessing was performed with MATLAB (2008a, the MathWorks, Natick, MA, USA) and Data Processing Assistant for Resting-State fMRI (DPARSF, Basic edition) [40]. The first 10 volumes were discarded for scanner calibration and adaption of participants to the circumstances. Other preprocesses included slice-timing, realignment, resampling to a 3 mm isotropic voxel size and spatial normalization with an EPI template. No participant’s data was discarded due to excessive head motion (more than 2.5 mm in translation or 2.5 degree in rotation).

A temporal filter (0.01 Hz < f < 0.08 Hz) was used to reduce very low-frequency drifts and physiological high-frequency noise [19]. Linear trends were removed. By using DPARSF, ReHo was calculated using the Kendall coefficient of concordance to measure the similarity of time courses of 27 neighboring voxels in a voxel-wise way: $W=\frac{\sum {\left(R_{i}\right)}^{2}-n{\left(\bar{R}\right)}^{2}}{\frac{1}{12}K^{2}\left(n^{3}-n\right)}$ where *W* is KCC among given voxels, ranging from 0 to 1. *R*_*i*_ is the sum rank of the *i*th time point; where *R* = ((n+1)/K)/2 is the mean of the *R*_*i*_’s; *K* is the number of time series within a measured cluster (here, *K* = 27, respectively; one given voxel plus the number of its neighbors); *n* is the number of ranks (here *n* = 78) [27]. Thus, the individual ReHo map of each dataset was generated. Then the individual ReHo map was smoothed with a Gaussian filter of 6 mm full width at half-maximum (FWHM). Finally, each ReHo map was divided by the global mean ReHo of each participant for standardization purposes as done in PET studies [41]. Finally, we obtained the smReHo for statistical analysis.

#### 2.3.2 Statistical analysis

A two-sample t-test was applied to compare the ReHo maps between schizophrenic patients with delusions and healthy controls. Multiple comparison correction was performed by using the AlphaSim program in REST 1.5 [42] (http://www.restfmri.net). The AlphaSim program of REST 1.5 was from AFNI [42]. A combination of individual voxel’s p < 0.05 and cluster size > 6156 mm^3^ (228 voxels) was used, which corresponds to a corrected p < 0.05 (rmm = 5, whole brain mask). Finally, a correlation analysis between the ReHo map and scores of CDRS and PANSS-Delusion subscale, respectively, were performed in the patient group in a voxel-wise way within the clusters, showing significant between-group differences, while the age, gender and medication dosage (chlorpromazine equivalents) were taken as covariates. To correct for the medication effect, all antipsychotic drugs the patients were taking were translated into the chlorpromazine equivalent [43]. The chlorpromazine equivalent of each patient was taken as a covariant during correlation analysis [13]. We also performed a correlation analysis between ReHo and CDRS while taking age, gender, medication dosage (chlorpromazine equivalents) and total score of PANSS, which reflected the severity of schizophrenia, as covariates, to reduce severity effect. Multiple comparison correction was performed by using the AlphaSim program in REST 1.5 [42] (http://www.restfmri.net). A combination of single voxel’s p < 0.05 and cluster size > 351 mm^3^ (13 voxels) was used, which corresponded to a corrected p < 0.05 (rmm = 5. Brain regions that survived the criteria for two sample t-tests were selected as a mask).

## Results

**Fig 1:**
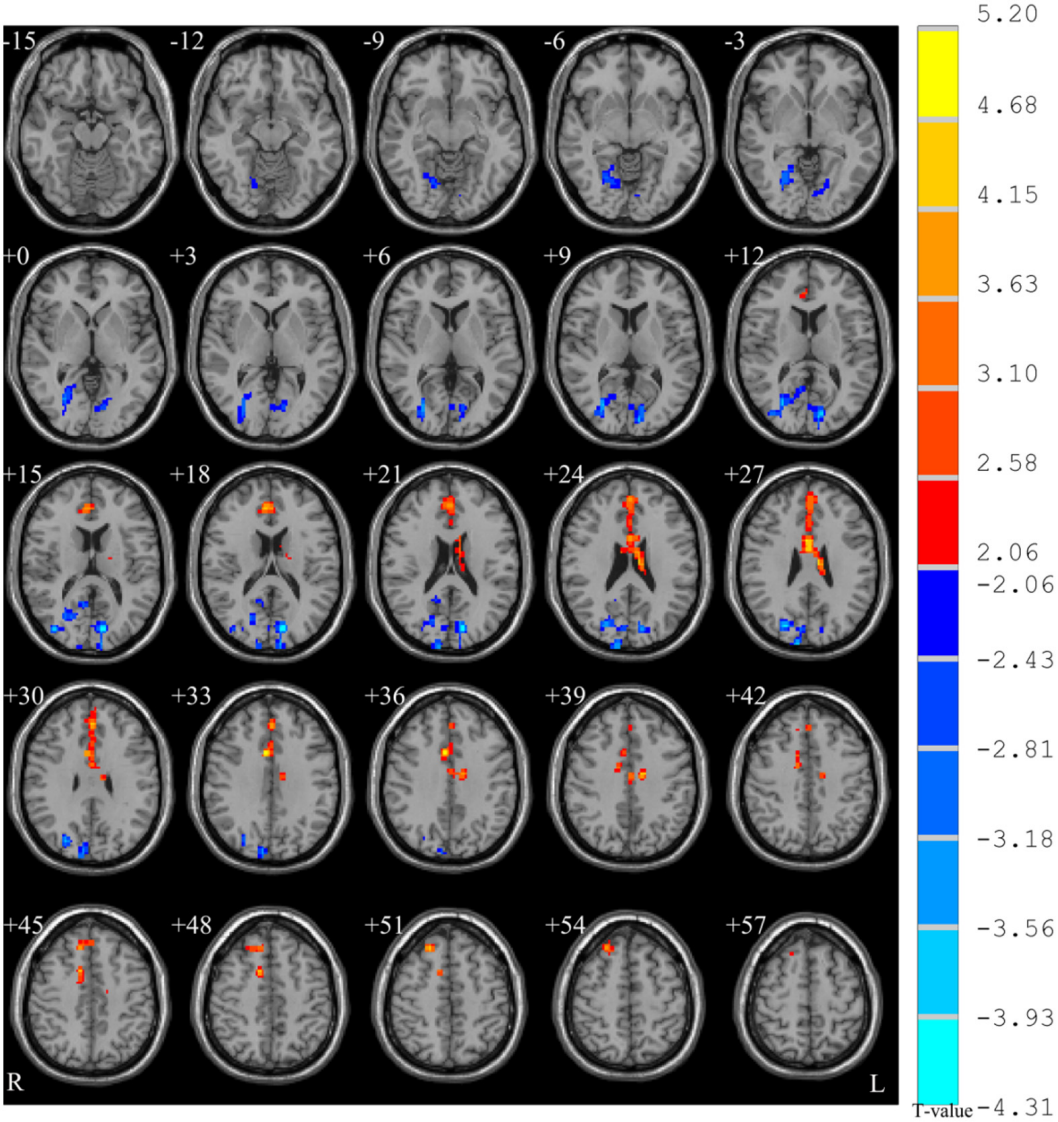
Two-sample t-test between groups (p < 0.05, corrected). Warm color indicates that ReHo is higher in the schizophrenic patient group than in the healthy control group, and vice versa.

**Fig 2:**
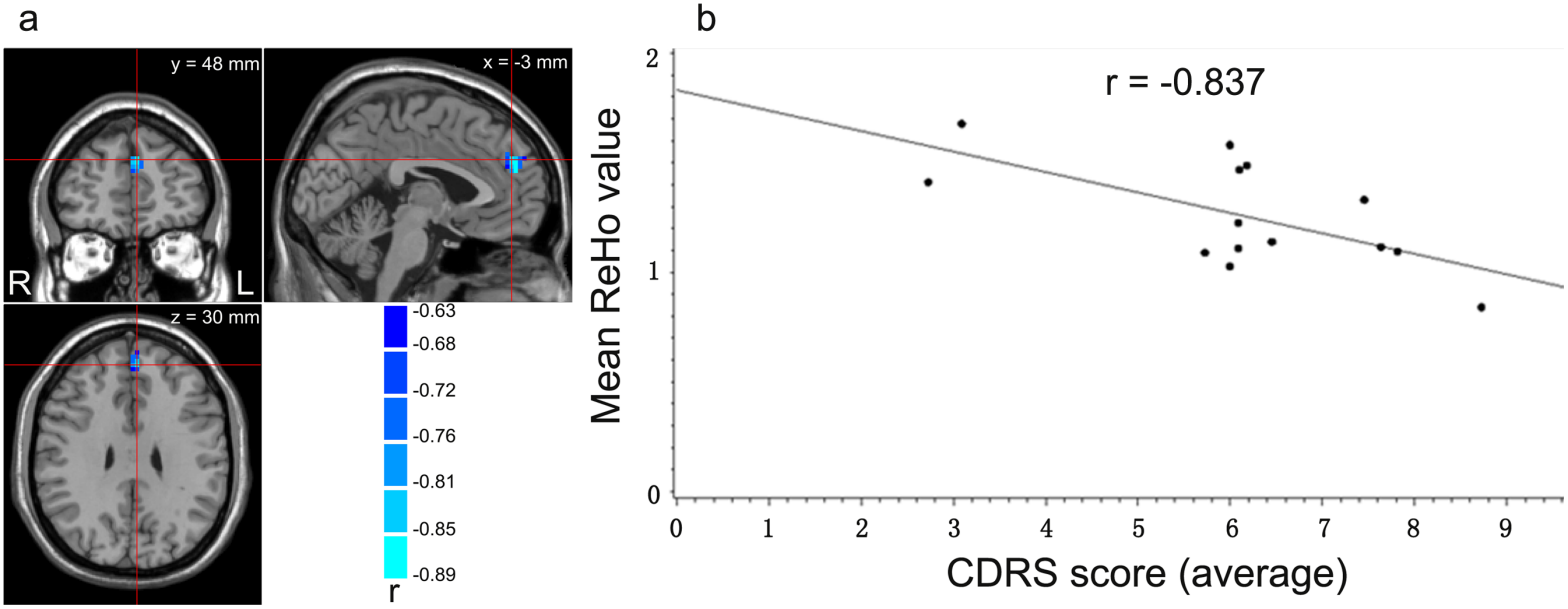
Results of correlation analysis (p < 0.05, corrected), controlling for medication. (a) The left medial superior frontal gyrus (mSFG; 918 mm^3^, 34 voxels, with peak coordinates at [-3, 48, 30] in the Montreal Neurology Institute system). (b) The plot of negative correlation between scores of CDRS and mean ReHo values across the cluster shown in (a) in schizophrenic patients with delusions.

Two sample t-tests (p < 0.05, corrected; see Fig 1 and Table 2) showed that ReHo in the right anterior cingulate gyrus (ACC), extending to left medial superior frontal gyrus (SFG), was higher in the patients group than in controls, while ReHo in the left superior occipital gyrus was lower in the patient group. The medial SFG is also named the dorsal medial prefrontal cortex (dMPFC) [44, 45]. The correlation analysis (p < 0.05, AlphaSim corrected; see Fig 2a and 2b controlling for medication) showed that CDRS score was negatively correlated with the ReHo in the left medial SFG (BA 9). There was no difference between with and without controlling for medication in the correlation analysis results. The correlation between antipsychotic dose and severity of delusion symptoms was significant (r = 0.867, p < 0.001). No significant correlation was found between PANSS delusions subscale score and mean ReHo values within the mask, either with or without the total PANSS score as a covariate.

**Table 2:** Results of two-sample t-test between groups.

	Peak Coordinates (MNI)				
Brain region	x	y	z	t value	Volume (mm^3^)
R anterior cingulate gyrus, extending to left medial frontal gyrus (BA 24 and BA 9)	6	12	33	3.53	12771
L superior occipital gyrus (BA 18 and BA19)	-12	-78	21	-4.12	16362

R: Right. L: Left. BA: Brodmann area. MNI: Montreal Neurological Institute.

## Discussion

To the best of our knowledge, this is the first study analyzing the ReHo of spontaneous brain activity and delusions of schizophrenic patients by RS-fMRI. We found that ReHo in the right ACC, extending to left dMPFC, was higher in schizophrenic patients compared to controls, while ReHo in the left superior occipital gyrus was lower in the schizophrenic patients group. The ReHo of the left dMPFC (BA 9) was negatively correlated with CDRS scores. No significant correlation of ReHo with PANSS delusions subscale score was found.

The alteration of ACC and dMPFC in schizophrenic patients has been found in many studies. Decreased volume of the right ACC [46, 47] and left dMPFC [13] has been reported in schizophrenic patients. The right ACC and left dMPFC were activated by a task designed to evoke sensations similar to delusions of reference in schizophrenia patients experiencing prominent referencing delusions [48]. In the current study, the increased ReHo in schizophrenic patients indicated that an increased local synchronization of spontaneous activity may be related to delusions. A previous resting-state fMRI study on schizophrenic patients found decreased ReHo in the left medial frontal gyrus, (BA 2, MNI coordinates: -12, -45, -18) [31], which was close to the left dMPFC in the current study. One possible explanation for the seemingly contradictory results is a difference in the schizophrenic patients. In our study, all schizophrenic patients had delusions while in that study the delusion symptoms were not mentioned [31]. As schizophrenia is a highly heterogeneous disorder, it will be important for future studies to use the same method and similar patients to replicate previous results.

The dMPFC was a highly active region. DMPFC was a hyper-perfusion area related to delusion in deluded patients with dementia with Lewy bodies [49]. Activation of dMPFC was elicited from both schizophrenia itself and endorsement of a psychosis state in schizophrenic patients experiencing referential delusions [48]. A delusion formation hypothesis suggests that the delusions arise from the brain’s attempts to integrate the disorganized neural processes experienced by patients with schizophrenia [13]. ReHo also indicated neural functional synchronization in certain brain regions [27]. Higher ReHo in DMPFC may indicate more integration of disorganized neural processes, which facilitates the formation of delusions.

In our study, the ReHo in dMPFC showed a negative correlation with delusion severity. This result is counterintuitive. Controlling for antipsychotic medicine has been considered as one reason. However, the correlated brain region between the ReHo map and delusion has not changed significantly based on whether or not medication was controlled for. Therefore, controlling medication does not appear to lead to the phenomenon. Head motion may influence RS-fMRI result [50, 51]. Yet head motion was also excluded. An explanation of the phenomenon may be the delusion formation hypothesis. They argued that the delusions arose from the brain’s attempts to integrate the disorganized neural processes experienced by patients with schizophrenia [13]. Compared to normal controls, schizophrenic patients needed to integrate disorganized neural processes for delusion. Therefore, patients showed higher ReHo than controls in the deluded brain region. However, among the patients themselves, the severe symptoms meant dysfunction of the brain. The severe patients did not have enough ability to integrate disorganized neural process. Therefore, the more severe delusional schizophrenic patients showed a decreased ReHo.

In this study, we found decreased ReHo in the left superior occipital gyrus of schizophrenic patients. However, in this area there was no significant correlation between ReHo values and CDRS scores. This was consistent with previous studies. Liu and colleagues also found decreased ReHo in the left inferior and middle occipital gyrus [31]. It seems that the decreased ReHo in the occipital area may not be closely related to delusions. Rather, an abnormal ReHo in the occipital area may be a more general abnormality in schizophrenic patients.

No significant correlation was found between ReHo maps and the PANSS delusions subscale, but a significant correlation with CDRS was found in schizophrenic patients with delusions. CDRS measures more dimensions of delusion than PANSS. Garety believed that delusion contained 11 dimensions, which were conviction, preoccupation, interference, resistance, dismissibility, absurdity, self-evidentness, reassurance, worry, unhappiness, and pervasiveness [38, 52]. However, the delusion subscale in PANSS only contains one item [37, 53]. This scale can only rate the severity of delusion in the most apparent aspects, such as conviction [54] or bizarreness [1]. It is quite plausible the CDRS provides more intrinsic information about delusions, which can be expressed in brain activity.

A few limitations in this study should be addressed. First, the sample is relatively small, especially considering that delusion is a very complex syndrome. If there is a source for an fMRI dataset, we may do more work similar to Turner’s [55]. Second, all patients were taking medicine. Results from drug naïve schizophrenia patients with delusions would be more helpful in understanding the brain mechanism of delusions. Third, although we have used the total score of PANSS as a covariate to exclude the confounding effects of other symptoms, it could not exclude the effects of other symptoms completely. If there is a direct comparison between delusion-dominated patients and non-deluded patients in the future, the results may be more significant. This should be studied further in the future.

In summary, the increased ReHo demonstrated by RS-fMRI in schizophrenic patients with delusions in the dMPFC may suggest that increased local synchronization of spontaneous brain activity may underlie the delusions.
